# Perioperative Diaphragm Dysfunction

**DOI:** 10.3390/jcm13020519

**Published:** 2024-01-17

**Authors:** Jinge Hu, Ruijuan Guo, Huili Li, Hong Wen, Yun Wang

**Affiliations:** 1 Department of Anesthesiology, Beijing Friendship Hospital, Capital Medical University, Beijing 100050, China; hujinge@mail.ccmu.edu.cn (J.H.); guorj@mail.ccmu.edu.cn (R.G.); lily@mail.ccmu.edu.cn (H.L.); 2 Department of Anesthesiology, Beijing Chaoyang Hospital, Capital Medical University, Beijing 100020, China; wendoctorhong@sina.com

**Keywords:** diaphragm, dysfunction, diagnosis, perioperative management

## Abstract

Diaphragm Dysfunction (DD) is a respiratory disorder with multiple causes. Although both unilateral and bilateral DD could ultimately lead to respiratory failure, the former is more common. Increasing research has recently delved into perioperative diaphragm protection. It has been established that DD promotes atelectasis development by affecting lung and chest wall mechanics. Diaphragm function must be specifically assessed for clinicians to optimally select an anesthetic approach, prepare for adequate monitoring, and implement the perioperative plan. Recent technological advancements, including dynamic MRI, ultrasound, and esophageal manometry, have critically aided disease diagnosis and management. In this context, it is noteworthy that therapeutic approaches for DD vary depending on its etiology and include various interventions, either noninvasive or invasive, aimed at promoting diaphragm recruitment. This review aims to unravel alternative anesthetic and operative strategies that minimize postoperative dysfunction by elucidating the identification of patients at a higher risk of DD and procedures that could cause postoperative DD, facilitating the recognition and avoidance of anesthetic and surgical interventions likely to impair diaphragmatic function.

## 1. Introduction

**Figure 1:**
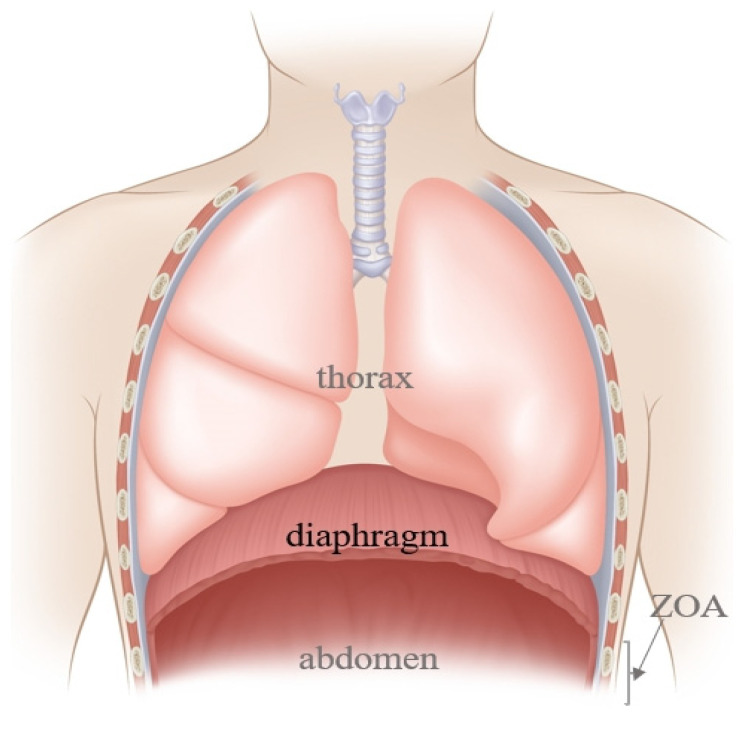
The anatomy of the diaphragm. ZOA denotes Zone of Apposition.

The diaphragm, a dome-shaped structure between the chest and the abdomen (Figure 1), contributes 60–80% of ventilation needs, making it a key driving force for respiratory movement [1]. Diaphragm Dysfunction (DD) is defined as multifactorial unilateral or bilateral diaphragmatic paralysis or long-term diaphragmatic weakening (partial loss of the pressure generation ability) and paralysis (total loss of diaphragmatic function) [2]. The disease could arise either from the weakness of the diaphragmatic muscle fiber or injury to its sole nerve supply, the phrenic nerve, which originates primarily from C4 but also receives contributions from the composition of C3 and C5 [3].

Based on the above overview, DD could be caused by disease processes that interfere with diaphragmatic innervation, contractile properties, or mechanical connections to the chest wall [4]. Furthermore, DD patients could exhibit varied clinical manifestations ranging from completely asymptomatic signs noticed incidentally on routine chest X-ray to permanent respiratory failure requiring long-term ventilator support, depending on the severity of the paralysis and whether the weakness is either unilateral or bilateral [5]. The potential complexity of this symptom presentation highlights the importance of active clinical management based on identifying risk factors and causes and implementing specific diagnostic and therapeutic approaches. This review aims to improve anesthetic management and prognosis of DD by providing a comprehensive overview of its perioperative period, including its clinical manifestations and causes, as well as diagnostic and therapeutic procedures, specifically focusing on perioperative management strategies commonly employed in clinical practice.

## 2. Manifestations and Clinical Characteristics of DD

Diaphragmatic weakness or paralysis could clinically affect either one or both hemidiaphragms. The natural history of DD is largely determined by its cause and the rate at which the underlying illness progresses. Age-related changes in respiratory drive, respiratory muscle strength, and chest-wall compliance may predispose individuals to hypoventilation [4]. Furthermore, unless there are comorbid conditions such as obesity and chronic pulmonary disorders [such as asthma, Chronic Obstructive Pulmonary Disease (COPD), or Obstructive Sleep Apnea (OSA)], the compromised respiratory function in individuals with Hemi-Diaphragmatic Paralysis (HDP), which is usually asymptomatic (often found incidentally upon routine chest X-ray examination, showing the elevation of one diaphragm), could be compensated for by the contralateral diaphragm and intercostal muscles [6]. On the other hand, most patients with bilateral involvement are generally more symptomatic. They have increased dyspnea and are particularly intolerant to the supine position. Furthermore, these patients often sleep in recliners and report fatigue and hypersomnia caused by sleep fragmentation and hypoventilation. These symptoms could partly be explained by the fact that the diaphragm is the only functioning inspiratory muscle during Rapid Eye Movement (REM) sleep [7,8,9]. Furthermore, patients with diaphragmatic paralysis are prone to pulmonary infections due to limited chest movement. Additionally, chronic hypoventilation could lead to hypoxia and hypercapnia, which are more frequent during sleep. Respiratory failure may result from severe respiratory muscle weakening. Patients may also develop the clinical features of right heart failure if the symptoms persist for a long time [10,11]. Moreover, a close association has been reported between DD and the onset and progression of several lung diseases. For instance, in COPD patients, a decline in diaphragm contraction function is closely related to respiratory difficulties and poor activity tolerance. In other words, diaphragm fatigue is critically involved in the development of respiratory failure in COPD patients [12]. Furthermore, the significant correlation between DD and pulmonary complications after thoracic surgery could contribute to hypoxemia, bronchospasm, and atelectasis [13].

The incidence of DD is yet to be accurately established and may vary depending on the study population and diagnostic method used. For example, depending on the ultrasound technique applied, DD incidence among dyspneic patients in emergency departments may range between 22.4 and 31.7% [14]. Additionally, DD prevalence was reported to exceed 60% (at admission) in critically ill patients requiring intubation, and was as high as 80% in patients experiencing difficult weaning, requiring prolonged Mechanical Ventilation (MV) [15]. Notably, the mortality rate of DD patients in the Intensive Care Unit (ICU) is also significantly higher (as high as 50%) than that of those without DD (16%). Therefore, DD is associated with MV prolongation, a higher failure rate of ventilator withdrawal, and a higher mortality rate [16]. Phrenic nerve lesions are well-known complications of cardiothoracic surgery [17]. Following cardiac surgery, DD incidence ranges from 1–60%, with 8% of patients developing persistent DD, which is associated with adverse respiratory outcomes [18]. A prospective research study revealed a high prevalence (68%) of postoperative DD after lung resection on the operated side [19]. Additionally, various regional anesthesia techniques may, more or less significantly, affect diaphragm function.

## 3. Diseases and Risk Factors Causing DD

Table 1 shows the causes of diaphragmatic dysfunction. The risk factors include obesity, cardiopulmonary comorbidities [20], preexisting neuropathies, history of cardiothoracic surgery, and hypothermia in cardiac surgery [21]. The causes can be clinically categorized as follows:

### 3.1. Patient-Related Factors (Underlying Diseases)

Patient-related causes of DD can be broadly categorized as follows: trauma (including thoracic penetrating traumas or gunshot injuries directly affecting the diaphragm and cervical nerve root injury), compression (including cervical spondylosis [22], substernal goiter [23], and aortic aneurysm), neurological diseases (including stroke [24,25]), myopathy, and neuromuscular junction diseases such as severe myasthenia [26]), connective tissue diseases (such as systemic lupus erythematosus), infections, electrolyte imbalances, diaphragmatic hernias, and cancers.

Any space-occupying lesion in the thoracic cavity adjacent to the phrenic nerve (such as mediastinal or pulmonary mass) could cause phrenic nerve injury via direct infiltration or external compression. Bilateral phrenic nerve palsy, which is secondary to a benign substernal goiter, may present as an acute respiratory failure requiring intubation or tracheostomy. In stroke patients, the thickness and motility of the bilateral diaphragm are decreased, with DD being more severe on the hemiplegic side. On the other hand, infections include bacterial toxins or viruses, and the mechanism underlying the sepsis-induced neuromuscular damage may be similar to that of sepsis-induced cardiomyopathy. The two potential mechanisms underlying the former are an alteration in blood flow to the diaphragm resulting from a reduced ability to extract and use energy substrates and a change in the diaphragm’s contractile capacity (weakness) [15]. Furthermore, a COVID-19-related HDP case, resulting in breathing difficulties, suggested that SARS-CoV-2 may cause phrenic neuropathy. Direct invasion of nerves or inflammatory effects are the two processes that could explain this phenomenon [27].

### 3.2. Anesthesia-Related Factors

Anesthesia-related factors include anesthetics (such as sedatives [28], opioids, and muscle relaxants), MV, and regional anesthesia (including the cervical plexus block, brachial plexus block, and high thoracic paravertebral nerve blocks).

Excessive sedation, neuromuscular blockade, excessive ventilator assistance leading to disuse atrophy, and centripetal load injury induced by excessive inspiratory effort and insufficient respiratory assistance are presently the four possible mechanisms for diaphragmatic injury. Ventilator-Induced Diaphragm Dysfunction (VIDD) occurs in up to 80% of MV patients. According to some animal and human studies, patients developed a certain degree of atrophy within 12–24 h after MV and deteriorated over time, with 50% unable to recover diaphragm activity within 24 h. Furthermore, patients undergoing shoulder and arm surgery often receive nerve blocks to help with pain control either preoperatively or postoperatively. Some regional anesthesia techniques often lead to diaphragmatic paralysis, given that the phrenic nerve runs in the fascia over the anterior scalene muscle. Urmey et al. discovered that the interscalene block led to an 100% unilateral phrenic nerve block, resulting in a 27% and 26.4% decrease in Force Vital Capacity (FVC) and Forced Expiratory Volume (FEV) in one second, respectively. This effect can be tolerated in healthy individuals rather than patients with pulmonary disorders [29]. Furthermore, the use of hypnotics, opioids, and steroids is associated with changes in diaphragm function. These changes may be attributed to the sedation-induced reduction in respiratory drive [30].

### 3.3. Surgery-Related Factors

Surgery-related factors include pneumoperitoneum, Trendelenburg positioning, and iatrogenic injuries (such as injuries induced by cardiothoracic vascular surgery, cervical and mediastinal tumor surgery, cervical spine surgery, and esophageal surgery). Laparoscopy has long been the standard surgical intervention in general surgery and gynecology. During surgery, prolonged pneumoperitoneum pressure and Trendelenburg positioning push the diaphragm towards the head, resulting in decreased lung compliance and Functional Residual Capacity (FRC) [31]. Iatrogenic phrenic nerve injury-induced DD is a well-recognized complication following cardiothoracic surgery [32,33]. Phrenic nerve injury may occur during lung cancer resection when the upper mediastinal lymph nodes are removed or when the hilum of the lung is moved closer to the phrenic nerve. The incidence rate after upper lobectomy (8.9%) is almost twice that following lower lobectomy (5.5%) [34]. Phrenic nerve palsy is a very common occurrence after cardiac bypass surgery. Since the phrenic nerve is located very close to the heart, the cold cardioplegia could cause phrenic nerve cold injury [35]. Phrenic nerve injury and diaphragmatic weakness are risks of mediastinal procedures, esophageal surgeries, or lung transplantation.

## 4. Common Diagnostic Methods for Perioperative DD

Selecting an appropriate screening and diagnosis approach for a specific clinical scenario is critical in evaluating suspected DD patients. Currently, there are multiple tools for evaluating diaphragm function and activity. Indirect techniques include chest X-ray, fluoroscopy, pulmonary function test, Computed Tomography (CT), and Magnetic Resonance Imaging (MRI). On the other hand, direct approaches include ultrasound, trans-diaphragmatic pressure (Pdi), and Electromyography (EMG).

### 4.1. Chest X-ray

**Figure 2:**
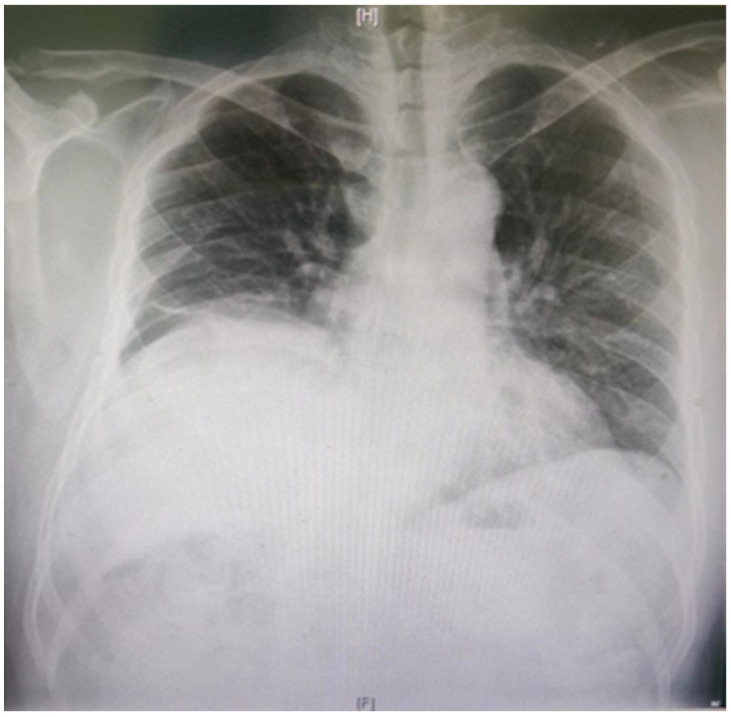
The chest radiograph shows the pathological elevation of the right hemidiaphragm.

In normal individuals, the left hemidiaphragm is usually located one intercostal space lower than the right hemidiaphragm. The negative pressure inside the chest will push the hemidiaphragm into the chest cavity if it is weak. Therefore, a paralyzed hemidiaphragm is always at a higher level (Figure 2). Although an elevated hemidiaphragm, as shown on a chest radiograph, is often a sign contributing to the diagnosis of unilateral paralysis, a similar radiographic appearance could also be observed in cases of diaphragmatic eventration, subpulmonic effusion, lobar atelectasis, or subphrenic abscess [36]. The chest X-ray diagnosis of DD has a high sensitivity and low specificity of 90% and 44%, respectively [37].

### 4.2. Fluoroscopic Examination

In normal breathing, the diaphragm descends by at least one intercostal space due to its contraction, with a more rapid and pronounced decrease during deep breathing or inhalation. On the other hand, the paralyzed diaphragm often shows no or abnormal movement during the sniffing test. Fluoroscopic examinations have a false positive rate of approximately 6% [38] and require substantial effort and cooperation from patients. Furthermore, ultrasonography is gradually replacing this test, given the health effects of ionizing radiation.

### 4.3. Pulmonary Function Test

The diaphragm provides 80% of respiratory muscle strength. In this regard, the pulmonary function test can easily detect DD. Common indicators examined in this test include Vital Capacity (VC), FVC, Total Lung Capacity (TLC), FRC, and Residual Volume (RV). This test should be performed in both sitting and supine positions for all suspected diaphragmatic paralysis patients. As evidenced by a decrease in FVC, diaphragmatic paralysis is associated with restrictive ventilation [39]. In a sitting position, the FVC of HDP and Bilateral Diaphragmatic Paralysis (BDP) patients is expected to decrease by 30% and 75%, respectively. Notably, restrictive dysfunction is more severe in the supine position. In the supine position, VC accounts for 70–80% and 30–50% of the predicted value in unilateral and bilateral paralysis, respectively. Furthermore, FRC and RV are usually normal in HDP patients [4] and decreased in BDP patients. Notably, the specificity of the pulmonary function test is low and is affected by patients’ compatibility. Therefore, it should be employed along with other detection methods to comprehensively evaluate diaphragmatic function.

### 4.4. CT and MRI

All patients with diaphragmatic weakness should be subjected to a CT or MRI examination to rule out any chest illnesses that may cause compressive damage to the phrenic nerve. According to research, CT and static MRI scans performed on subjects with different lung volumes provide useful information on the surface area and positioning of the whole diaphragm within the thorax [36]. In addition to correlating changes in diaphragm position with changes in lung volume, these images are useful in elucidating the role of the diaphragm in pulmonary disease conditions [40,41]. Furthermore, when sufficiently detailed, these images could assist in detecting clinically important changes in the thickness of the muscle itself. Therefore, CT and static MRI are useful tools in assessing diaphragm atrophy. Moreover, as an emerging technique, dynamic MRI can give information on the contribution of DD to pulmonary impairment, which has been used to evaluate Diaphragm Excursion (DE) in COPD patients [42,43].

### 4.5. Ultrasound

Diaphragmatic Ultrasound (DUS) is highly sensitive (93%) and specific (100%) in diagnosing DD [44]. It allows for the assessment of DE, diaphragmatic thickness, and thickening in time or over time, especially in ambulatory and MV patients. Furthermore, this technique can assist in identifying disease processes underlying DD, as well as inform clinical decision-making and guide postoperative function exercise. Nevertheless, the ultrasound-based DD definition is not standardized, and diagnosis methods may vary. Furthermore, DUS parameters cannot replace standard weaning indices or clinical judgment.

Two modes of ultrasonography, B-mode and M-mode, can be employed for disease diagnosis. While the former detects diaphragmatic thickness (Tdi) and real-time echogenicity, the latter displays the diaphragm’s movement curve and evaluates DE direction, amplitude, and rate in a certain area over a specific period. In patients with diaphragm weakness and paralysis, DUS can demonstrate abnormalities in motion, thickness, and thickening. Notably, further exploration and optimization are required for emerging ultrasonography imaging technologies, including tissue Doppler imaging [45], shear wave ultrasound elastography, and speckle tracking imaging.

#### 4.5.1. DE

Diaphragm excursion refers to the diaphragmatic displacement between the end of inspiration and the end of expiration. Diaphragm function has been assessed through DE post-surgery [46], as well as in critically ill patients requiring MV [47] and patients with neuromuscular disorders [48]. Boussuges et al. reported that the normal DE values in healthy adults during normal and deep breathing were (1.8 ± 0.3) and (7.0 ± 0.6) cm for males and (1.6 ± 0.3) and (5.7 ± 1.0) cm for females, respectively [49]. Postoperative DD assessed through DUS is defined as a DE < 10 mm or negative. This cutoff value has been validated in different patients, including healthy volunteers, postoperatively in patients undergoing abdominal surgery, and critically ill patients [49,50,51]. The association between DE and Diaphragmatic Thickening Fraction (DTF) has been reported to be very weak [52]. Notably, DE is unsuitable for MV as some of the pressure conveyed by MV causes passive diaphragmatic displacement, which cannot reflect diaphragmatic contraction and function [51]. Moreover, DE has not been validated as a DD index as it depends on multiple factors.

#### 4.5.2. Tdi

**Figure 3:**
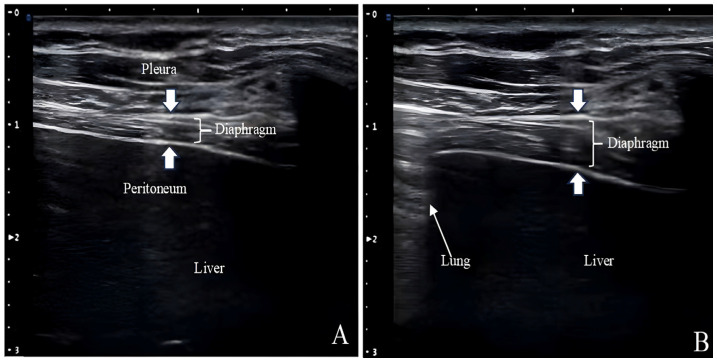
Measurement of diaphragmatic thickness through B-Mode ultrasonography. The distance between the pleura and peritoneum represents diaphragmatic thickness. The images show a change in normal diaphragm thickness between end-exhalation (**A**) and end-inspiration (**B**). The lung is visualized at end-inspiration.

Ultrasound measurements of Tdi have been used to assess diaphragm atrophy in patients with neuromuscular disorders and those requiring MV (Figure 3) [53,54]. The Tdi during inhalation is comparable to the cardiac ejection fraction and is a reliable respiratory effort indicator [55]. The lower Tdi limit for normal adults is 0.80–1.60 mm, and posture, lung volume, and selected intercostal space can affect the measurements [36]. Given that Tdi is measured in millimeters off the ultrasound screen with a cursor of a certain thickness and from a tracing that may not be perfectly outlined, it is susceptible to the “small number effect”.

#### 4.5.3. DTF

The diaphragm shortens and thickens during contraction. This thickening can be quantified based on DTF using the following formula: change in thickness from end-exhalation to peak-inhalation/thickness at end-exhalation × 100. The “ABCDE” technique directly assesses Tdi through the intercostal space without requiring specific acoustic windows involving the liver or spleen [56]. According to research, DTF is a good inspiratory effort indicator in the diaphragm and a good predictor of weaning outcomes [57]. A DTF <20% indicates diaphragmatic paralysis [58]. As a surrogate measurement of the intensity of voluntary diaphragm contraction, DTF has been used in phrenic neuropathy patients [58] and critically ill patients [59]. Nevertheless, some studies reported high inter-individual variability in the relationship between DTF and changes in airway pressure [60] and Pdi or the diaphragm’s electrical activity [61].

### 4.6. Pdi (cm/H_2_O) 

For Pdi measurement, a balloon catheter should be inserted through the nose into the lower esophagus and stomach, followed by the calculation of the pressure difference between the stomach and esophagus. Clinically, Pdi could be measured during tidal breathing, maximum sniff maneuvers (sniff Pdi), maximum inspiratory efforts against a closed glottis (Pdi max), and transcutaneous electrical or magnetic stimulation of the phrenic nerve (twitch Pdi). The sniff Pdi or Pdi max in males and females is >80 and >70, respectively. Clinically significant diaphragmatic weakness is excluded if the twitch Pdi is >10 during unilateral phrenic nerve stimulation and if Pdi is >20 during bilateral phrenic nerve stimulation [9]. Notably, although Pdi measurement is usually the gold standard for BDP diagnosis, it involves invasive tests which could cause patients discomfort.

### 4.7. Diaphragmatic Electromyography (Diaphragmatic EMG) 

Esophageal diaphragmatic EMG and surface diaphragmatic EMG are the two main methods for detecting EMG. The former is recorded from the crural diaphragm using a multi-pair esophageal electrode catheter, reducing contamination from the EMG of other respiratory muscles. However, the insertion of the catheter into the esophagus limits its use throughout the perioperative period. On the other hand, the latter is recorded with surface electrodes placed in the skin of the respiratory muscles, which may be affected by subcutaneous fat thickness, electrode placement, and peripheral interference, among other factors [62]. Notably, diaphragmatic EMG can distinguish between neurological and myopathic dysfunction [4]. Although surface diaphragmatic EMG is currently the gold standard for detecting synchronies in noninvasive ventilation, it is not always effective and cannot be used for routine bedside tests. Furthermore, diaphragmatic EMG indices are inferior to DUS in predicting the MV liberation outcome [63]. Overall, the perioperative application of diaphragmatic EMG is limited due to technological challenges and complexities in its interpretation. 

Overall, each detection technique has its strengths and limitations. In summary, although chest X-ray, CT imaging, and static MRI generate still images of the diaphragm, thereby providing information about the shape and position of the muscle, they cannot provide direct information on DE and diaphragm function. On the other hand, fluoroscopy can give misleading results, especially when assessing patients with HDP or bilateral paralysis. Additionally, although dynamic MRI has great potential, allowing for the study of the diaphragm’s motion of segments in multiple planes, it is not widely available. Furthermore, technological challenges and complexities in interpreting diaphragmatic EMG limit its perioperative application. Additionally, although DUS has gained popularity in the last decade for being noninvasive, simple to operate, and portable, it is highly operator-dependent and position-dependent. Finally, whether CT and MRI are superior to DUS in diagnosing diaphragm weakness or paralysis remains to be determined.

## 5. Perioperative Management Strategies for DD Patients

The etiologies and clinical presentations of DD are varied, thereby complicating its perioperative management, which involves addressing distinct clinical problems at three phases of care: before, during, and after surgery. Furthermore, DD could be discovered at presentation, induced during operation, or exacerbated in recovery. The combined effects of DD occasionally accrue post-surgery and may lead to transient or persistent respiratory failure. Decisions leading to the operation, operative and anesthesia steps, and perioperative use of medications should be promptly analyzed to prevent DD progression.

### 5.1. Preoperative Management

A thorough examination may reveal diaphragmatic weakness in some patients. Upon encountering signs of muscular weakness at any site, a close preoperative examination of respiratory muscle function should be performed. According to studies, M-mode ultrasonography is an effective preoperative and postoperative bedside method for screening diaphragmatic paralysis [64]. Furthermore, optimizing patients’ status before surgery is fundamental for those with a preexisting weakness. An example in this regard is expiratory muscle strength training for four weeks in patients with neurodegenerative diseases [65]. It has also been reported that noninvasive ventilation and mechanical cough assist devices can help with airway clearance and lung inflation, and lower the risk of atelectasis [66,67]. Patients who regularly use these devices should continue using them throughout the perioperative phase [68].

### 5.2. Intraoperative Management

It is critical to recognize, reduce, and, if possible, avoid anesthetic and surgical interventions that may impair diaphragmatic function. Here, we explored MV, regional block, and anesthetics as the primary aspects of intraoperative management.

#### 5.2.1. MV

Both the lung and the diaphragm could be damaged by MV. While the importance of lung-protective ventilation is well-established, the concept of diaphragm-protective ventilation is a significant but still unproven new paradigm. A protective ventilation strategy for the diaphragm should be implemented, including avoiding insufficient diaphragm activity, excessive diaphragm activity, and patient-ventilator asynchrony, all of which could be targeted by specific ventilation strategies, possibly mitigating the occurrence or severity of diaphragm myotrauma. All these strategies require strengthening diaphragmatic function monitoring during the perioperative period and selecting an appropriate ventilator technique [69]. 

An optimal inspiratory effort level comparable to that of healthy subjects at rest may protect diaphragms and improve clinical outcomes [70]. In this regard, maintaining appropriate diaphragm activity during MV could prevent diaphragmatic injury. According to research, providing assisted MV rather than controlled MV could reduce muscle protein hydrolysis and weakness [71,72]. Furthermore, transitioning a clinically stable patient to spontaneous breathing using assisted ventilation modes with reduced or interrupted sedation might preserve diaphragm activity and prevent the occurrence of disuse atrophy [73]. Proportional modes of ventilation support, like Neurally Adjusted Ventilatory Assist (NAVA) and Proportional Assist Ventilation (PAV), enable patients’ control-of-breathing system to regulate ventilation and limit over- and under-assist, thereby enhancing diaphragm recovery [74]. Patient-ventilator asynchrony poses a significant challenge to the protective ventilation of the lungs and diaphragms. Patient-ventilator asynchrony can be identified by carefully examining the ventilator waveform, as well as adjusting ventilator settings and monitoring muscle relaxation in patients with general anesthesia. Muscle relaxants could be added promptly per the surgical progress. Furthermore, it is noteworthy that lung protection takes precedence over diaphragm protection when there is a conflict in choosing between them. Nonetheless, further research is required to evaluate how both the diaphragm and lung protective targets could be synchronized into a single ventilation strategy.

#### 5.2.2. Regional Blocks

Regional blocking technology as the main anesthesia method can protect physiological diaphragmatic function and reduce intraoperative atelectasis in patients with spontaneous breath, especially those with risk factors such as asthma, COPD, and OSA [75]. However, general anesthesia is usually used along with regional blocks for upper limb surgery, especially the shoulder. Proximal brachial plexus blockades, such as interscalene and supraclavicular blocks, have been linked to HDP. However, the clinical value of providing effective analgesia or surgical anesthesia using a nerve block should be evaluated against the potential risk of phrenic nerve paresis.

Although the interscalene block [which anesthetizes the C5 and C6 nerve roots by depositing Local Anesthetics (LAs) between them] is the most common regional anesthetic technique, its utility among patients at high risk of respiratory complications is limited due to the high incidence of ipsilateral phrenic nerve blockade (virtually 100%) [29]. Multiple novel diaphragm-sparing regional techniques currently available include the upper trunk block, combined suprascapular and axillary nerve block, combined infraclavicular and suprascapular nerve block, and costoclavicular brachial plexus block (Table 2). These techniques allow for selective anesthetization of nerves necessary for inducing analgesia for shoulder surgery while preserving diaphragmatic function [20]. Moreover, recent research highlights the importance of employing specific strategies to reduce phrenic nerve paralysis, including limiting LA doses and volume, and strategically administering injections away from the C5-C6 nerve roots [76,77]. On the other hand, administering ultra-low volume and doses of LAs will reduce the duration of analgesia. Therefore, a promising solution is using intravenous dexamethasone or perineural LA adjuvants that prolong the duration of both sensory-motor blockade and analgesia.

Cervical Plexus Blocks (CPBs), which have been used to facilitate various procedures, such as carotid endarterectomy and clavicular or thyroid surgery, can be classified as superficial, intermediate, and deep [97]. The effect of LAs on diaphragm function remains controversial, especially regarding whether they can penetrate the prevertebral fascia and then block the phrenic nerve. Using 20 mL of 0.5% ropivacaine, Opperer et al. studied the characteristics and side effects of three CPBs and found that DD was most pronounced in the deep CPB group [98]. Furthermore, Han et al. used different concentrations of LAs (10 mL, either 0.3% or 0.5%) for intermediate CPB, and found that both could induce DD on the block side (29% vs. 58%, respectively) at 40 min, with a comparable incidence (46% vs. 65%, respectively) at 4 h post-block [99]. On the other hand, intermediate CPB induced using 0.2 mL/kg of 0.25% ropivacaine did not cause ipsilateral HDP [100]. The significant difference in HDP incidence in the above results may be associated with the volume of LAs and injection rate, among other possible factors.

#### 5.2.3. Anesthetics

A protocol that avoids using muscle relaxants and reduces the use of perioperative sedation and narcotics provides a model for managing patients with related neuromuscular weaknesses. Furthermore, halogenated anesthetic agents are appropriate for patients with motor neuron, peripheral nerve, and neuromuscular junction diseases. Conversely, halogenated agents should be avoided for patients with myopathies due to the increased risk of malignant hyperthermia or rhabdomyolysis. Total intravenous anesthesia is recommended for such patients. However, intravenous anesthetic agents and opioids could also cause further respiratory depression. The neuromuscular blockade can be prolonged in all patients with neuromuscular diseases. Train of Four monitoring and reversal with sugammadex are recommended if muscle relaxants must be used [68]. Appropriate anesthetics are required to maintain adequate sedation during prolonged MV, especially in the ICU. Compared to dexmedetomidine or propofol, midazolam results in lower antioxidant activity and a higher lipid peroxidation and protein ubiquitination level. Furthermore, during MV, sedation with midazolam worsens diaphragm function compared to dexmedetomidine and propofol [101].

### 5.3. Postoperative Management

Shortening the duration of unnecessary MV through extubation immediately after surgery can generally accelerate the postoperative recovery of diaphragm function. It is important to consider residual neuromuscular blockade (a major cause of DD in patients who receive general anesthesia, which can be related to aspects of medication administration or patient factors) when a patient unexpectedly shows respiratory weakness and inadequate ventilation immediately after surgery. The HDP of high-risk patients receiving regional anesthesia must be monitored postoperatively. If the patient cannot be extubated postoperatively and is transferred to the ICU, the focus should be on extubating as soon and safely as possible. If the duration of MV exceeds 24–48 h in patients with preexisting diaphragmatic weaknesses, early tracheostomy should be considered to facilitate spontaneous ventilation to achieve independent respiration [64]. Further treatments should be considered if DD recovery takes too long.

## 6. Treatment

The treatment of DD patients depends on the etiological diagnosis, the presence or absence of symptomatic complaints, and nocturnal hypoventilation. In cases of potentially reversible paralysis with a known cause, specific therapeutic options are available. For asymptomatic patients with HDP without underlying cardiopulmonary conditions, prognosis is considered good and no intervention should be administered. However, for symptomatic patients, especially for high-risk patients or when the condition is more acute with correctable causes, other treatment options should be used. Treatment measures include observation, ventilatory support, rehabilitation treatment, diaphragm pacing (DP), and surgical intervention (e.g., diaphragmatic plication and phrenic nerve reconstruction).

Before treatment, coexisting diseases that could potentially affect respiratory function, such as obesity, COPD, and metabolic disorders, should be fully corrected. In some cases, a wait-and-observe approach should be adopted, for example, in cases of cooling from cardiac surgeries or after a nerve block suspected of causing weakness [17]. 

While both unilateral and bilateral diaphragmatic paralysis benefit from respiratory support, the spectrum of options evolves with disease progression. Non-invasive positive pressure ventilation emerges as a valuable non-surgical tool for managing bilateral diaphragm weakness, delivering both clinical and blood gas improvements in the long term [102]. However, as neuromuscular conditions advance, most patients ultimately require MV, which can be delivered non-invasively (nasal masks) or invasively (tracheostomy) [58]. Encouragingly, even after various conditions like COPD, spinal cord injury, or bypass surgery, early rehabilitation with techniques like inspiratory muscle training can still enhance diaphragmatic function in patients with paralysis [103].

The technical and clinical success of DP is dependent on adequate phrenic nerve function. Prospective candidates for DP should be free from significant lung diseases or primary muscle diseases [104]. It is effective in patients with high cervical spinal cord injury and central hypoventilation [9]. The induced contraction of the diaphragm by pacing the phrenic nerve has the potential to not only reduce the rate of its atrophy during MV but, in fact, probably increase Tdi [105]. Therefore, DP may represent a promising approach for maintaining diaphragm strength.

Among the series of patients operated on, the main causes of paralysis are trauma and iatrogenic injuries [102]. Diaphragmatic plication involves folding the paralyzed diaphragm, so that it is immobilized in a position of maximum inspiration, thereby relieving compression of the lung parenchyma. Moreover, it is primarily indicated for patients with selective HDP (patients with severe breathing difficulties, cough or chest pain, or ventilator dependence). For patients with diaphragm paralysis, plication offers a safe and minimally invasive option to alleviate symptoms and improve breathing. While patients with morbid obesity or progressive neuromuscular diseases may require alternative approaches [106], plication shines in its ability to improve diaphragm function. However, for those seeking complete restoration of diaphragmatic movement, phrenic nerve reconstruction offers a targeted approach, directly addressing nerve damage and overcoming anatomical hurdles [107].

## 7. Conclusions

Strong evidence demonstrates that DD is a frequent and serious clinical concern in critically ill patients. Much attention should be paid to asymptomatic HDP to obtain a detailed medical history, identify high-risk patients, and clarify causes during preoperative evaluation in a timely manner. For patients with unilateral or bilateral diaphragmatic paralysis, an appropriate perioperative anesthesia management plan should be established. Prior to upper limb surgery, a careful evaluation of pulmonary function is essential, particularly for potential diaphragmatic paralysis on the contralateral side, due to the variable and non-negligible risk of HDP associated with regional anesthesia techniques. Furthermore, postoperative monitoring of HDP is crucial for high-risk patients with pre-existing respiratory comorbidities. Ultrasound is widely applied in the diagnosis of DD due to its noninvasiveness, repeatability, and validity. In the context of Enhanced Recovery after Surgery, a protective ventilation strategy that targets the lung and diaphragm should integrate various factors, optimize respiratory effort, monitor respiratory dynamics, and prevent phrenic atrophy and injury, thereby improving the prognosis of patients.

## Figures and Tables

**Table 1:** Causes of diaphragmatic dysfunction.

Patient-Related Factors	Anesthesia-Related Factors	Surgery-Related Factors
trauma	anesthetics	pneumoperitoneum
compression	upper limb regional blocks	Trendelenburg position
neurological and neuromuscular junction diseases	mechanical ventilation	iatrogenic injuries
myopathy		
connective tissue diseases		
diaphragmatic hernia		
cancers		

**Table 2:** Summary of common blocking techniques for shoulder and upper limb surgery.

Types	Procedures	Full Surgical Anesthesia?	Incidence of HDP *	Study
STB	Shoulder surgery	Yes	4.8–76.3%	Kim et al., 2019 [78] Robles et al., 2022 [79] Lee et al., 2021 [80] Kang et al., 2019 [81]
SCB	Upper limb surgery	To be determined	0–68%	Renes et al., 2009 [82] Aliste et al., 2018 [83] Kang et al., 2018 [84] Oh et al., 2020 [85] Wang et al., 2023 [86] Petrar et al., 2015 [87] Bao et al., 2019 [88]
CCB	Upper limb surgery, shoulder surgery	Yes	0–11.4%	Sivashanmugam et al., 2019 [89] Aliste et al., 2019 [90] Oh et al., 2020 [85] Hong et al., 2021 [91]
ASSNB	Shoulder surgery	No	4–20%	Doğan et al., 2022 [92] Sehmbi et al., 2019 [93]
ICB + SSNB	Shoulder surgery	Yes	0–5.6%	Aliste et al., 2018 [94] Taha et al., 2019 [95]
SSNB + ANB	Shoulder surgery	No	2–41%	Ferré et al., 2020 [96]

STB = Superior Trunk Block, SCB = supraclavicular block, CCB = Costco-clavicular block, ASSNB = anterior suprascapular nerve block, ICB + SSNB = combined infraclavicular and suprascapular nerve blocks, SSNB + ANB = combined suprascapular and axillary nerve blocks. HDP = hemi-diaphragmatic paralysis. * based on RCT or cadaveric dye study data.

## Data Availability

Not applicable.
